# Associations between alcohol use and accelerated biological ageing

**DOI:** 10.1111/adb.13100

**Published:** 2021-10-12

**Authors:** Sunniva M.K. Bøstrand, Kadi Vaher, Laura de Nooij, Matthew A. Harris, James H. Cole, Simon R. Cox, Riccardo E. Marioni, Daniel L. McCartney, Rosie M. Walker, Andrew M. McIntosh, Kathryn L. Evans, Heather C. Whalley, Robyn E. Wootton, Toni-Kim Clarke

**Affiliations:** 1 Division of Psychiatry, Royal Edinburgh Hospital, The University of Edinburgh, Edinburgh, UK; 2 Centre for Regenerative Medicine, Institute for Regeneration and Repair, The University of Edinburgh, Edinburgh, UK; 3 MRC Centre for Reproductive Health, The Queen’s Medical Research Institute, The University of Edinburgh, Edinburgh, UK; 4 Centre for Medical Image Computing, Department of Computer Science, University College London, London, UK; 5 Institute of Psychiatry, Psychology and Neuroscience, King’s College London, London, UK; 6 Dementia Research Centre, Institute of Neurology, University College London, London, UK; 7 Department of Psychology, The University of Edinburgh, Edinburgh, UK; 8 Centre for Genomic and Experimental Medicine, Institute of Genetics and Molecular Medicine, The University of Edinburgh, Edinburgh, UK; 9 Centre for Clinical Brain Sciences, The University of Edinburgh, Edinburgh, UK; 10 MRC Integrative Epidemiology Unit, University of Bristol, Bristol, UK; 11 School of Psychological Science, University of Bristol, Bristol, UK

**Keywords:** alcohol use, brain ageing, epigenetic ageing, Generation Scotland, Mendelian randomisation, UK Biobank

## Abstract

Harmful alcohol use is a leading cause of premature death and is associated with age-related disease. Biological ageing is highly variable between individuals and may deviate from chronological ageing, suggesting that biomarkers of biological ageing (derived from DNA methylation or brain structural measures) may be clinically relevant. Here, we investigated the relationships between alcohol phenotypes and both brain and DNA methylation age estimates. First, using data from UK Biobank and Generation Scotland, we tested the association between alcohol consumption (units/week) or hazardous use (Alcohol Use Disorders Identification Test [AUDIT] scores) and accelerated brain and epigenetic ageing in 20,258 and 8051 individuals, respectively. Second, we used Mendelian randomisation (MR) to test for a causal effect of alcohol consumption levels and alcohol use disorder (AUD) on biological ageing. Alcohol use showed a consistent positive association with higher predicted brain age (AUDIT-C: β = 0.053, *p* = 3.16 × 10^−13^; AUDIT-P: β = 0.052, *p* = 1.6 ×10^−13^; total AUDIT score: *β* = 0.062, *p* = 5.52 × 10^−16^; units/week: *β* = 0.078, *p* = 2.20 × 10^−16^), and two DNA methylation-based estimates of ageing, GrimAge (units/week: *β* = 0.053, *p* = 1.48 × 10^−7^) and PhenoAge (units/week: *β* = 0.077, *p* = 2.18×10^−10^). MR analyses revealed limited evidence for a causal effect of AUD on accelerated brain ageing (β = 0.118, *p* =0.044). However, this result should be interpreted cautiously as the significant effect was driven by a single genetic variant. We found no evidence for a causal effect of alcohol consumption levels on accelerated biological ageing. Future studies investigating the mechanisms associating alcohol use with accelerated biological ageing are warranted.

## 1 Introduction

Harmful alcohol use is a leading cause for premature death globally.^1^ Excessive alcohol use affects multiple tissues^2^ and is associated with an increased risk for all-cause mortality^3^ and age-related diseases including diabetes, liver diseases and dementia.^4^ A recent large-scale epidemiological investigation has challenged the view that moderate alcohol consumption can be beneficial, showing that even small amounts of alcohol negatively impact on health.^1^ Ageing itself is a complex process of progressive deterioration due to the accrual of cellular damage over time,^5^ and rates of these age-associated biological processes vary between individuals. This could account for some of the variation in susceptibility to age-related disease and suggests that measures of biological ageing may be more clinically relevant than chronological age.^6,7^ Several biomarkers have been proposed to measure individual variation in biological ageing, including those based on DNA methylation (DNAm) or brain magnetic resonance imaging (MRI). To date, studies investigating associations between alcohol use and biological ageing have been limited to small sample sizes, report inconsistent findings and have not probed the potential causality of these associations.

Recent analytical approaches, such as that developed by Cole et al.,^6^ use machine learning algorithms trained to predict chronological age from brain structural MRI data. Testing these algorithms on new structural data produces a metric of brain age that correlates strongly with chronological age, although its deviation from chronological age reflects accelerated/decelerated brain ageing.^8^ Accelerated brain ageing predicts mortality in older adults and correlates with cognitive and physical decline.^9^ Several epigenetic clocks have also been used to characterise biological ageing and are hypothesised to capture molecular processes involved in declining tissue function.^10^ These biomarkers use weighted averages of methylation levels at specific cytosine-phosphate-guanine (CpG) sites to produce estimates of epigenetic age. Similarly to brain age, a greater positive deviation in DNAm age from chronological age predicts all-cause mortality^11^ and has been linked to a range of age- and lifestyle-related health outcomes, including exercise and diet,^12^ and cognitive ability and decline.^13,14^

Previous work has associated alcohol use with the acceleration of biological ageing, as measured by both brain and DNAm ageing.^12,15–19^ Ning et al.^15^ showed that more frequent consumption of alcohol was associated with a higher brain age relative to peers, with the lowest brain age found in individuals who reported drinking only occasionally. Higher alcohol intake frequency was also associated with an older-appearing brain using multiple MRI modalities to predict brain age.^16^ Similarly, higher levels of alcohol consumption across 30 years of follow-up were associated with reductions in grey matter density and white matter structural integrity, suggesting that heavy alcohol consumption may lead to accelerated brain ageing.^2^

Alcohol use is associated with variation in DNAm^20^; thus, investigations into the associations between alcohol use and epigenetic ageing could allow insight into the shared molecular mechanisms underlying harmful alcohol use and ageing. Previous studies have reported complex relationships between alcohol consumption levels and epigenetic ageing, with studies showing positive,^18^ negative^12^ and non-linear^19^ associations. Additionally, a recent study suggests both positive and negative genetic associations between various alcohol-related traits and epigenetic ageing using LD score regression.^21^

Cross-sectional, observational epidemiological studies are prone to bias from residual confounding and reverse causation, limiting the investigations of cause−effect relationships. Genome-wide association studies (GWASs) have identified several loci implicated in both the clinical diagnosis of alcohol use disorder (AUD) and varying alcohol consumption levels.^22,23^ These genetic variants can be utilised as instruments to determine the most likely direction of effect between a modifiable exposure, alcohol use and the outcome, biological ageing, using Mendelian randomisation (MR).^24^ It follows the logic that if a modifiable exposure (e.g. alcohol use) is the cause of an outcome (e.g. accelerated biological ageing), then individuals with genetic variants predisposing them towards increased alcohol use should be more likely to experience accelerated biological ageing.

Here, we investigated the relationship between alcohol use and biological ageing using the largest brain imaging (*N* = 20,258) and DNAm (*N* = 8051) datasets to date. We hypothesised that higher levels of alcohol consumption would associate with both higher DNAm age and brain age and that these associations would reflect a causal effect of higher alcohol use on accelerated biological ageing.

## 2 Methods and Materials

The UK Biobank (UKB) and the Generation Scotland: Scottish Family Health Study (GS:SFHS) cohort data were used in the current study. For a graphical representation of the samples and data available in both of the cohorts for the current analyses, see Figure S1.

### 2.1 Study populations: UKB

UKB comprises *N* = 502,617 individuals recruited from across the United Kingdom.^25^ UKB received ethical approval from the National Health Service (NHS) National Research Ethics Service North West (reference: 11/NW/0382). The present study was carried out under UKB project ID 4844. At the time of writing, we used the latest available UKB neuroimaging (see protocol in Miller et al.^25^) release consisting of *N* = 21,386 individuals. After removing extreme outliers (defined as MRI measurements >5*SD from the mean), cases of image acquisition problems and excluding previous or never drinkers, *N* = 20,258 individuals were included in the present study (see Table S1 and Figure S2 for demographics and sample selection).

#### 2.1.1 Alcohol consumption

Lifestyle measures were collected at baseline and online follow-up. Alcohol use in units/week at baseline was calculated by converting the sum of reported average weekly intake of red wine, champagne plus white wine, beer plus cider, spirits, fortified wine and other alcoholic drinks into alcohol units. At online follow-up, a subset of participants (*N* = 14,710) completed the Alcohol Use Disorders Identification Test (AUDIT), a 10-item screening tool developed by the World Health Organisation^26^ to assess alcohol consumption and alcohol-related behaviours and problems. To examine the relationship between alcohol use and brain age, we used four measures of alcohol consumption: alcohol units/week and three measures from the AUDIT questionnaire—a composite score of alcohol consumption (AUDIT-C; sum of Questions 1−3), problematic alcohol use (AUDIT-P; sum of Questions 4−10) and the total score across all items (AUDIT-T).

#### 2.1.2 Brain age estimates

We utilised a measure of brain age derived from structural T1-weighted MRI data as described by Cole et al.^6^ and implemented using the brainageR software package (https://github.com/james-cole/brainageR). In the sample of current drinkers, the correlation between brain age and chronological age was *r* =0.734, *p* < 2.2 × 10^−16^ (full demographics in Table S1). This measure was subsequently residualised over chronological age, sex, imaging site (Manchester/Newcastle) and scanner head position X, Y and Z coordinates. The residualised brain age measure reflected deviation of brain age from chronological age (controlling for aforementioned covariates), with positive values representing accelerated brain ageing.

### 2.2 Study populations: Generation Scotland

GS:SFHS cohort comprises ~24,000 individuals aged ≥18 years at recruitment and is described in detail elsewhere.^27^ At baseline, participants were assessed for a range of health, demographic and lifestyle factors and provided samples for DNA extraction. GS:SFHS has been granted ethical approval from the NHS Tayside Committee on Medical Research Ethics, on behalf of the NHS (reference: 05/S1401/89) and has Research Tissue Bank status (reference: 15/ES/0040). The present study includes individuals for whom information about alcohol consumption, smoking, body mass index (BMI) and DNAm data (profiled in two sets; see below) was acquired at baseline (full demographics in Table S2).

#### 2.2.1 Alcohol consumption

We used self-reported units/week as a quantification of a person’s alcohol consumption. The present study includes individuals who reported being current drinkers; extreme outliers (defined as participants with alcohol consumption in units/week >4* standard deviations [SD] from the mean) were additionally excluded from the analysis. For a comparison between the GS:SFHS participants included and not included in the current study, see Table S14.

#### 2.2.2 DNAm profiling

Whole blood genomic DNA samples were treated with sodium bisulphite using the EZ-96 DNA Methylation Kit (Zymo Research, Irvine, California), following the manufacturer’s instructions. Genome-wide DNAm was profiled using the Infinium MethylationEPIC BeadChip (Illumina Inc., San Diego, California) in accordance with the manufacturer’s protocol. DNAm was profiled in 9778 participants across two processing sets (Set 1 *N* = 5190 [comprised related individuals], Set 2 *N* = 4583 [comprised unrelated (to each other and to Set 1) individuals]). Quality control and normalisation was carried out separately in the two sets using standard methods. Arrays were scanned using a HiScan scanner (Illumina Inc., San Diego, California), with initial inspection of array quality carried out in GenomeStudio v2011.1. Additional quality control measures were implemented as described in detail previously.^28^ Briefly, outlier sites and participants, together with participants for whom there was a mismatch between their predicted sex (based on DNAm data) and their recorded sex, were excluded from both sets; samples were then normalised (separately) using the Dasen method from the watermelon^29^ R package. The final DNAm dataset comprised data for 5087 individuals in Set 1 and 4450 individuals in Set 2. Participant data in the two sets were analysed separately and then meta-analysed. After applying exclusion criteria (see above), the current study included 4260 participants from Set 1 and 3791 participants from Set 2 (full demographics in Table S2).

#### 2.2.3 Epigenetic estimates of age

Epigenetic age and age acceleration measures were calculated using the online age calculator (https://dnamage.genetics.ucla.edu/) developed by Horvath.^10^ Normalised DNAm beta-values were submitted to the calculator using the ‘Advanced Analysis for Blood Data’ option. Four DNAm-based estimates of age were calculated, namely, Hannum^30^ and Horvath^10^ epigenetic age, DNAm GrimAge^31^ and DNAm PhenoAge^32^ (see below), which all strongly correlated with chronological age (Table S3) and with one another (Table S15). From these estimates, four age-adjusted epigenetic age acceleration (EAA) measures (intrinsic and extrinsic EAA [IEAA and EEAA, respectively],^33^ AgeAccelGrim^31^ and AgeAccelPheno^32^) were calculated (see below). The age acceleration measures were uncorrelated with chronological age (Table S3). In addition, the resulting EAA measures were only weakly to moderately correlated with one another (Table S15), suggesting they capture different aspects of DNAm ageing.

The Horvath epigenetic age was calculated based on methylation levels at 353 CpG sites following the approach developed by Horvath.^10^ Hannum epigenetic age was calculated based on DNAm levels at the 71 CpGs identified by Hannum.^30^ From these DNAm age estimates, we derived two measures for epigenetic age acceleration that are either independent of blood cell counts or enhanced by changes in blood cell composition, as described previously.^33^ In brief, intrinsic epigenetic age acceleration (IEAA) is defined as the residual term of a multivariate model regression estimated Horvath methylation age on chronological age, adjusting for blood cell counts estimated from the methylation data (naive CD8+ T cells, exhausted CD8+ T cells, plasmablasts, CD4+ T cells, natural killer cells, monocytes and granulocytes), and is therefore by definition independent of blood immune cell counts. Extrinsic epigenetic age acceleration (EEAA), on the other hand, tracks age-related changes in blood cell composition as well as cell-intrinsic epigenetic changes. It is estimated by first calculating Hannum’s DNAm age and second increasing the contribution of three cell types whose abundance is known to change with age (naive cytotoxic T cells, exhausted cytotoxic T cells and plasmablasts) by forming a weighted average of Hannum’s DNAm age estimate with these three cell type estimates using the Klemera−Doubal approach.^34^ EEAA is defined as the residual term of univariate model regressing the weighted estimated Hannum’s epigenetic age in chronological age.

The DNAm GrimAge was developed by Lu et al.^31^ by first creating DNAm-based surrogates for 12 plasma proteins and smoking pack-years and thereafter regressing time to death on chronological age, sex and these DNAm-based surrogates. This model selected chronological age, sex and DNAm-based surrogates for smoking pack-years and seven plasma proteins (adrenomedullin, beta-2-microglobulin, cystatin C, growth differentiation factor 15, leptin, plasminogen activation inhibitor 1 and tissue inhibitor metalloproteinase). The linear combination of these variables is then used to estimate the DNAm GrimAge. Adjusting DNAm GrimAge for chronological age generated the measure of epigenetic GrimAge acceleration, AgeAccelGrim.^31^

The DNAm PhenoAge was developed by Levine et al.^32^ by first regressing ageing-related mortality on 42 clinical markers and chronological age to select variables for inclusion in the phenotypic age (PhenoAge) score. Ten variables (chronological age, albumin, creatinine, glucose and C-reactive protein levels, lymphocyte percentage, mean cell volume, red blood cell distribution width, alkaline phosphatase and white blood cell count) were then used to calculate mortality score, which was subsequently converted into units of years to create the measure of PhenoAge. Thereafter, DNAm from whole blood was used to predict this PhenoAge. This approach generated the selection of 513 CpGs, and linear combination of the weighted CpGs yields the DNAm PhenoAge.^32^ Adjusting DNAm PhenoAge for chronological age generated the measure of phenotypic epigenetic age acceleration, AgeAccelPheno.

### 2.3 Statistical methods

All statistical analyses were performed in R (Versions 3.3.2, 3.6.1 and 4.0.1).^35^ Scaling by z-transformation was applied for all numeric variables in the regression models.

#### 2.3.1 Association between alcohol use and brain age in UKB

The variables AUDIT-C, AUDIT-P, AUDIT total scores and alcohol consumption in units/week were entered separately into linear models to test for association with residualised brain age. Smoking status (coded at baseline as a binary value denoting whether individuals had ever smoked or never smoked), age and sex were added as covariates in each model in order to control for the effects of these variables. As a sensitivity analysis, we additionally adjusted for age of completing full-time education in the regression model (Figure S3). Benjamini−Hochberg correction for false discovery rate (FDR) was applied to the regression models.

#### 2.3.2 Association between alcohol consumption and epigenetic age acceleration in GS:SFHS

Set 1 data (related subset of GS:SFHS) statistical analyses were conducted in ASReml-R Version 3.0 (www.vsni.co.uk/software/asreml) to fit a linear mixed model to control for relatedness within the sample by fitting an inverse relationship matrix derived from pedigree information as a random effect. Set 2 data (unrelated subset of GS:SFHS) was analysed using linear regression (lm) function in base R. In each model, EAA was fit as the dependent variable, and alcohol consumption (as log_10_[1 + units/week] to adjust for non-normal distribution) as the independent variable; sex, BMI, smoking pack-years (at baseline, individuals were asked to self-report their tobacco exposure [cigarettes/day], age when they started smoking and years since stopped smoking, and pack-years variable was calculated as packs [20 cigarettes/pack] smoked per day multiplied by years as a smoker) and inverse relationship matrix fitted as a random effect only for Set 1 in ASReml were added as covariates. A sensitivity analysis was conducted by additionally adjusting the models for years of education (this information is collected from GS:SFHS participants in 10 categories corresponding to 0, 1−4, 5−9, 10−11, 12−13, 14−15, 16−17, 18−19, 20−21, 22−23, or 24+ years of education). As smoking is strongly associated with DNAm, further sensitivity analyses were conducted in non-smoking individuals (i.e. who reported never having smoked tobacco): *n* = 2207 in Set 1 and *n* = 1998 in Set 2. To combine the coefficient estimates from the two sets into a single estimate, we applied an inverse variance-weighted fixed-effects meta-analysis model using the function metagen, implemented in the meta package in R.^36^ FDR correction for multiple testing was applied across all fully adjusted models (fully adjusted regressions in Sets 1 and 2 and meta-analysis) and separately for the two sensitivity analyses. Results were plotted using the function forest in the R package meta.

### 2.4 Two-sample MR

Two-sample MR analysis was performed in R using the *TwoSampleMR* package from MRBase^37^ using summary statistics extracted from non-overlapping GWASs.

#### 2.4.1 Exposure GWAS: Alcohol consumption (AUDIT-C) and AUD

Data on the genetic association with alcohol use were extracted from Kranzler et al.^22^ This study was carried out in the Million Veteran Programme population, which is based in the United States and therefore highly unlikely to overlap with UKB or GS:SFHS populations made up of British and Scottish individuals, respectively. Kranzler et al.^22^ reported a genome-wide significant association between 10 single nucleotide polymorphisms (SNPs) and AUD, as well as 13 SNPs associated with alcohol consumption as measured by AUDIT-C. These SNPs were identified as independent by linkage disequilibrium (LD) clumping using a 500-kb genomic window and *r*^2^ < 0.1.^22^ Here, we extracted the summary statistics as reported in the European ancestry population (see Tables S1 and S2 in Kranzler et al.^22^).

#### 2.4.2 Outcome GWASs

##### Brain age

GWAS for brain age acceleration was performed in a subset of unrelated White British individuals in the UKB imaging cohort. 16,133 individuals were included using a relatedness cut-off of 0.05. To perform the GWAS, brain age was entered as the outcome variable and age, sex, genotyping array and the 20 first principal components derived from genotype data as covariates. From the results of this analysis, we extracted the summary statistics (beta and standard error of the effect allele of each SNP) for the 10 and 13 SNPs identified as genome-wide significant by Kranzler et al.^22^ for AUD and AUDIT-C, respectively. The full GWAS summary statistics are available here: https://datashare.is.ed.ac.uk/handle/10283/3797. We used the Functional Mapping and Annotation of Genome-Wide Association Studies (FUMA)^38^ SNP2GENE function to extract results from the brain age GWAS. In order to identify independently associated variants, clumpbased pruning was applied in FUMA using an *r*^2^ of 0.1 and a 1-Mb sliding window using the UKB White British sample as the LD reference panel. See Table S17 for the 20 top hits and Figure S6 for a Manhattan plot of main results. Using the GWAS summary statistics, we calculated the SNP-based heritability (Table S18) and genetic over-lap between brain age and ~770 other disease traits (Table S19) using LD score regression implemented in the online software LD Hub (http://ldsc.broadinstitute.org/).^39^

##### Epigenetic age

Data on the genetic association with AgeAccelGrim and AgeAccelPheno were extracted from McCartney et al.^21^ These GWASs were conducted in 34,962 European ancestry individuals, and a fixed-effects meta-analysis was performed to combine the summary statistics.^21^ Supplementary tables were inspected to ensure that the Million Veteran Programme cohort was not included in these meta-GWASs. From the GWAS results (available here: https://datashare.is.ed.ac.uk/handle/10283/3645), we extracted the corresponding summary statistics for the 13 SNPs identified as GW significant for AUDIT-C by Kranzler et al.^22^

For SNPs unavailable in the outcome GWAS summary statistics, proxy SNPs were searched for (https://ldlink.nci.nih.gov/?tab=ldproxy) with a minimum LD *R*^2^ = 0.9. In AgeAccelGrim and AgeAccelPheno GWASs, rs185177474 was not available; thus, rs151242810 was used as a proxy (*R*^2^ = 0.976).

See Tables S4 and S5 for the full MR input testing causal effects of AUDIT-C and AUD, respectively, on biological ageing.

We performed two MR analyses with brain age as the outcome, where AUD or AUDIT-C were the exposures, respectively. One MR analysis was performed with either AgeAccelGrim or AgeAccelPheno as the outcome and AUDIT-C as the exposure. The main MR models included 13 SNPs to probe for the causal effect of AUDIT-C on biological ageing and 10 SNPs to probe for the causal effect of AUD on brain ageing.

We applied complementary two-sample MR methods (inverse variance weighted [IVW], MR-Egger, weighted median and weighted mode-based estimation). IVW was the main analysis with each of the others providing sensitivity analyses, which each make different assumptions about the nature of pleiotropy (where the genetic variant associates with the outcome via an independent pathway to the exposure). Therefore, the strongest evidence for a causal effect would be where the estimates from all methods are consistent. To test the suitability of the MR-Egger method, the *I*^2^ statistic was calculated to quantify the degree of regression dilution bias due to measurement error of SNP-exposure effects.^40^ The mean *F*-statistic as an indicator of instrument strength was also calculated (Table S7). We additionally calculated the variance explained by the genetic instruments of AUDIT-C and AUD using a modified method as described in^41^ (Table S20). Further, we used the MR-Egger intercept to test for the presence of horizontal pleiotropy (Table S9) and Steiger filtering^42^ to test for the most likely direction of effect (Tables S10−S13) and calculated Cochran’s *Q* to assess heterogeneity suggestive of pleiotropy (Table S8). When there was evidence for a causal effect based on the IVW model, we performed MR-Presso^43^ and Radial MR^44^ to detect potential outliers.

## 3 Results

### 3.1 Alcohol use associations with brain age in UKB

**Figure 1:**
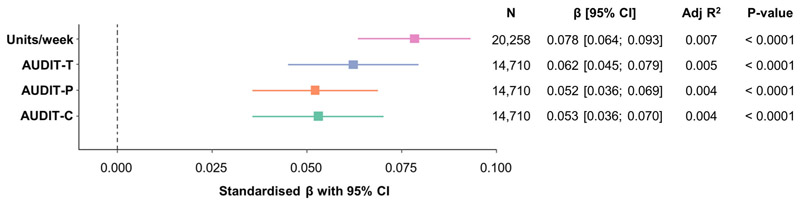
Alcohol use is associated with advanced brain age. Linear regression models predicting residual brain age from AUDIT-C, AUDIT-P, AUDIT-T and alcohol units, in current drinkers adjusted for smoking status. Plot shows standardised β coefficients with 95% confidence intervals. CI, confidence interval

Using linear regression, we found a consistent positive relationship between brain age and the four measures of alcohol use (alcohol units/week (*N* = 20,258, 52.1% female), AUDIT-C, AUDIT-P and AUDIT-T (*N* = 14,710, 53.5% female)) (Figure 1), with higher levels of self-reported alcohol consumption associated with a more advanced brain age. The largest effect was found for alcohol consumption measured in units/week (β = 0.078, 95% CI [0.066; 0.093], *p* < 2.20 × 10^−16^). Adjusting for education did not significantly alter the outcome of this analysis for any measure of alcohol use (Figure S3).

### 3.2 Alcohol use associations with epigenetic age in GS:SFHS

**Figure 2:**
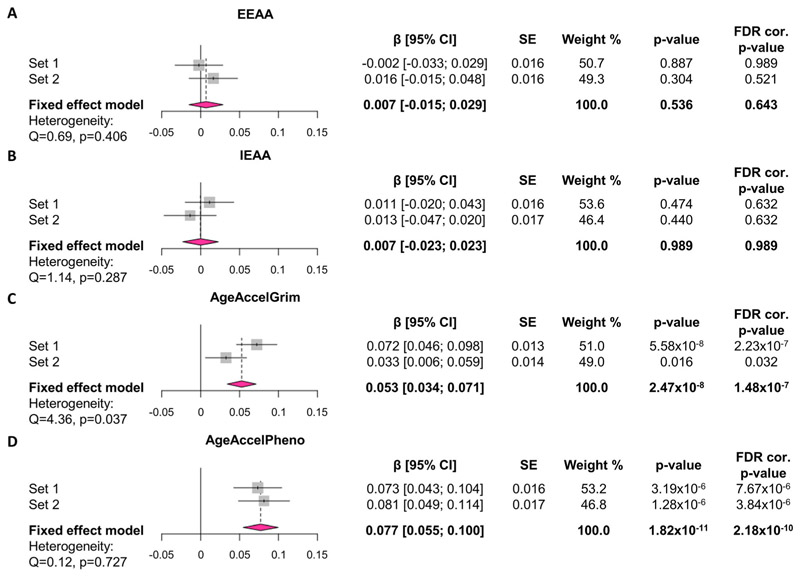
Alcohol consumption is associated with two measures of advanced epigenetic age. Effects of alcohol consumption (units/week) on (A) EEAA, (B) IEAA, (C) AgeAccelGrim and (D) AgeAccelPheno in fully adjusted models. Values on forest plot indicate standardised β with 95% confidence intervals. Models are adjusted for sex, BMI and pack-years in Sets 1 and 2 and relatedness in Set 1 by fitting pedigree information as a random effect in general linear mixed models using advanced restricted maximum likelihood (ASReml) method. Fixed-effects inverse variance-weighted meta-analysis was applied using R package *meta* to combine the standardised coefficient estimates in Sets 1 and 2. FDR correction was applied across all models in Sets 1 and 2 and all meta-analysis models (12 models in total). Sample size: *n* = 4260 in Set 1, *n* = 3791 in Set 2 (*n* = 8051 included in meta-analyses). CI, confidence interval; EEAA, extrinsic epigenetic age acceleration; FDR, false discovery rate; IEAA, intrinsic epigenetic age acceleration; SE, standard error

Linear regression and fixed-effects meta-analyses were used to investigate associations between alcohol consumption (units/week) and four measures of EAA (IEAA, EEAA, AgeAccelGrim and AgeAccelPheno) in 8051 individuals in total (Set 1 *n* = 4260 [60.9% female], Set 2 *n* = 3791 [55.3% female]) from the GS:SFHS cohort (full demographics in Table S2). We found a positive association between alcohol consumption and AgeAccelGrim (β = 0.053 [0.034; 0.071], *p* = 1.48 × 10^−7^) and AgeAccelPheno (β = 0.077 [0.055; 0.100], *p* = 2.18 × 10^−10^), but not between alcohol consumption and IEAA or EEAA (Figure 2). These results are robust to adjustment for years of education (Figure S4).

**Figure 3:**
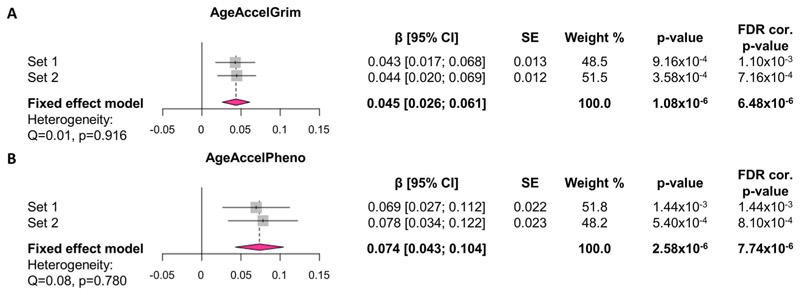
Alcohol consumption is associated with advanced GrimAge and PhenoAge in non-smokers. Effects of alcohol consumption (units/week) on (A) AgeAccelGrim and (B) AgeAccelPheno in non-smoking participants. Values on forest plot indicate standardised β with 95% confidence intervals. Models are adjusted for sex and BMI in Sets 1 and 2 and relatedness in Set 1 by fitting pedigree information as a random effect in general linear mixed models using advanced restricted maximum likelihood (ASReml) method. Fixed-effects inverse variance-weighted meta-analysis was applied using R package *meta* to combine the standardised coefficient estimates in Sets 1 and 2. FDR correction was applied across all smoking sensitivity models in Sets 1 and 2 and all meta-analysis models (six models in total). Sample size: *n* = 2207 in Set 1, *n* =1998 in Set 2 (*n* = 4205 included in meta-analyses). CI, confidence interval; EEAA, extrinsic epigenetic age acceleration; FDR, false discovery rate; IEAA, intrinsic epigenetic age acceleration; SE, standard error

As smoking is strongly associated with DNAm, we conducted a second sensitivity analysis by exploring the associations between alcohol consumption and AgeAccelGrim and AgeAccelPheno in a subset of non-smoking participants (Figure 3). The positive associations between alcohol consumption and the two EAA measures remained significant in non-smokers, but the effect size was slightly (~15%) attenuated for AgeAccelGrim (β = 0.045 [0.026; 0.061], *p* = 6.48 × 10^−6^) although it remained similar for AgeAccelPheno (β = 0.074 [0.043; 0.104], *p* = 7.74 × 10^−6^).

### 3.3 Testing for the causal influence of alcohol use on accelerated brain age

**Figure 4:**
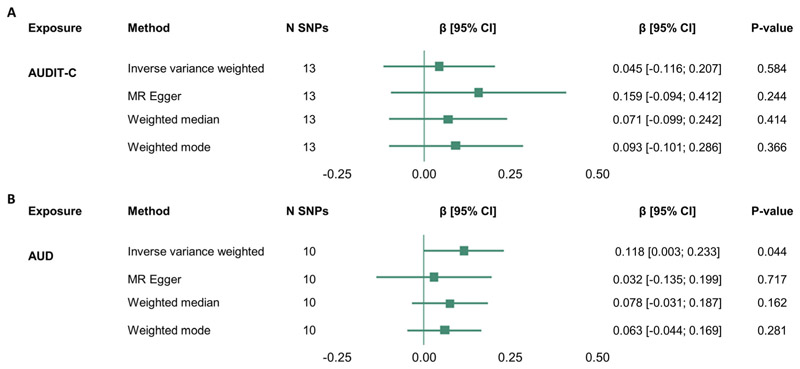
Two-sample Mendelian randomisation analysis provides weak evidence for a causal effect of AUD on brain age acceleration. (A) Two sample Mendelian randomisation of AUDIT-C on brain age. (B) Two-sample Mendelian randomisation of AUD on brain age. Data on the genetic association with AUDIT-C and AUD were extracted from Kranzler et al.^22^ Summary statistics for these SNPs were extracted from a novel GWAS of brain age (see Section 2). AUD, alcohol use disorder; CI, confidence interval; N SNP, number of SNPs included in the MR analysis

Having demonstrated a consistent phenotypic association between alcohol use and accelerated brain age, we used two-sample MR to test a causal relationship between AUD/alcohol consumption (AUDIT-C) and brain age (Figure 4). For AUD, the IVW model was significant (β = 0.118 [0.003; 0.233], *p* = 0.044; mF = 73.744 [Table S7]) (Figure 4B) but not for AUDIT-C (Figure 4A), suggesting that AUD, but not alcohol consumption levels, has a possible causal effect on accelerated/advanced brain age. However, Steiger filtering suggested that two of the genetic variants explained more variance in the outcome, suggesting some potential for reverse causation (Table S13). Additionally, Radial MR revealed rs570436 (*Q* = 4.854, *p* = 0.028) as an outlier in the MR analysis of AUD and brain age, and therefore, the analysis was repeated with this SNP excluded. After removing the outlying SNP, the IVW model was no longer significant (β = 0.092 [−0.001; 0.185], *p* =0.054), suggesting that the outlier was driving the significant result for AUD (Figure S5), although the biological function of this variant is not known, so we cannot be sure whether it acts through alcohol consumption. Together, these results provide limited evidence for a causal effect of genetically instrumented AUD on brain age and no evidence of a causal effect of alcohol use on brain age as measured by AUDIT-C.

### 3.4 Testing for the causal influence of alcohol consumption on epigenetic age acceleration

**Figure 5:**
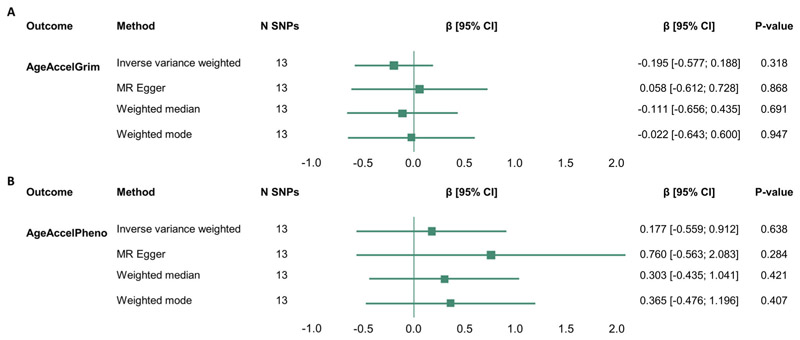
Two-sample Mendelian randomisation analysis shows no evidence for causal effect of alcohol consumption on measures of epigenetic age acceleration (A) AgeAccelGrim and (B) AgeAccelPheno. Data on the genetic association with alcohol use (AUDIT-C) were extracted from Kranzler et al.^22^ Summary statistics for these SNPs were extracted from GWASs for AgeAccelGrim and AgeAccelPheno conducted by McCartney et al.^21^ rs185177474 was not available in AgeAccelGrim and AgeAccelPheno summary statistics; thus, rs151242810 was used as a proxy (*R*^2^ = 0.976; see Section 2). CI, confidence interval; N SNP, number of SNPs included in the MR analysis

As we observed, a significant association between alcohol consumption and advanced GrimAge and PhenoAge in the observational analysis, we used two-sample MR methods to test whether these effects might be causal. Although the mean F-statistics suggest that the SNPs included in the analyses are strong genetic instruments (mF =79.058; Table S7), there was no evidence to suggest a causal effect of alcohol consumption (AUDIT-C) on the two EAA measures (Figure 5). Thus, these results show that in this study, we find no evidence that the association between alcohol consumption and accelerated epigenetic age is causal.

## 4 Discussion

This study represents one of the largest systematic investigations of alcohol use and biological ageing to date and is the first study investigating possible causal relationships between alcohol use and accelerated brain and epigenetic ageing using two-sample MR. We report consistent positive associations between four measures of alcohol use and accelerated brain age as well as alcohol consumption and two measures of DNAm age acceleration (AgeAccelPheno and AgeAccelGrim). MR analyses revealed limited evidence for the causal effect of AUD on accelerated brain age, although there was no evidence to suggest a causal link between levels of alcohol consumption and brain or epigenetic ageing.

In the present study, we demonstrate a positive association between four measures of alcohol use and a metric of accelerated brain ageing, derived from structural MRI. We show associations with problematic alcohol use (AUDIT-P), alcohol consumption (AUDIT-C) and total AUDIT scores, with the strongest phenotypic association found for alcohol consumption in units/week. These results are consistent with previous investigations showing that brain changes associated with ageing are more pronounced in individuals with higher levels of alcohol use^2^ and with previous reports of advanced brain age relative to peers in individuals who consume alcohol more frequently.^15,16^ The present study provides evidence that brain changes in response to excessive alcohol use resemble an early ageing process.

We further demonstrate a positive association between alcohol consumption and accelerated DNAm PhenoAge and GrimAge, thus expanding on the results of Fiorito et al.^18^ who showed positive association between alcohol consumption levels and accelerated PhenoAge. However, we do not replicate previous findings showing associations between self-reported alcohol consumption levels and accelerated DNAm age derived from Hannum and Horvath clocks,^12,18^ which could be attributed to differences in study populations,^12^ differences in the use of alcohol use variables in the models^18^ and the use of different DNAm arrays.^12,18^ Furthermore, the first-generation clocks (Horvath and Hannum) were designed for the Illumina 27 k and 450 k arrays, whereas PhenoAge and GrimAge were developed using overlapping CpG sites on the Illumina 450 K and EPIC arrays, and there are reports suggesting inaccurate Hannum and Horvath age estimations when using the EPIC array.^45^ Importantly, the novel PhenoAge and GrimAge estimators are shown to be stronger predictors of mortality and lifestyle factors, including alcohol use,^18,31,32^ which could be explained by the inclusion of CpG sites associated with biomarkers of physiological dysregulation, disease and mortality, whereas Hannum and Horvath clocks were designed to predict chronological age.

A major strength of the current study is the use of data from both UKB and GS:SFHS, which enabled association studies to be conducted in much larger samples from single cohorts compared with previous reports (*n* = 20,258 for brain ageing in the current study compared with *n* = 14,701,^16^ *n* = 12,115^15^ and *n* = 527^2^ in previous studies; *n* = 8051 for the DNAm ageing in the current study compared with *n* = 836,^19^ *n* = 3687^12^ and *n* = 16,245^18^ [from 18 different cohorts with each between 174 and 2817 participants] in previous studies). An additional strength is the range of measurements enabling a systematic assessment of the association of four different self-reported alcohol use measures with brain age acceleration as well as the association between alcohol consumption and four different measures of EAA.

Using two-sample MR, we report limited evidence suggesting a causal link between the diagnosis of AUD and accelerated brain ageing, but no evidence for higher alcohol consumption causing accelerated brain or epigenetic ageing. We replicate and expand on recent findings showing significant genetic correlations between alcohol-related phenotypes and epigenetic ageing, but no causal relationship between alcohol use frequency and EAA measures as assessed by MR.^21^ This suggests that the phenotypic association may arise from confounding factors (e.g. other harmful lifestyle factors) that have directional effects on both alcohol use and biological ageing. High levels of psychiatric comorbidity with AUD^46^ represent another possible confounder. For example, schizophrenia,^47^ major depressive disorder^48,49^ and post-traumatic stress disorder^50^ are associated with accelerated brain and/or epigenetic ageing. Furthermore, sensitivity analyses revealed that some of the genetic variants used here for alcohol use explain more variance in the outcome (brain or DNAm ageing), suggesting potential reverse causation. Finally, the Million Veteran Programme sample used for the GWAS of AUD and alcohol consumption comprises predominantly male armed forces veterans.^22^ Thus, it may not be representative of the whole population, as there is evidence that alcohol use and AUD have higher prevalence in males^1^ and genetic mechanisms might differ between sexes. Additionally, there are differences in the phenotypes used in the current study and the ones used in the GWAS for AUD and alcohol consumption by Kranzler et al.^22^ (e.g. clinically diagnosed AUD vs AUDIT-P). Future longitudinal studies combined with more experimental approaches could help elucidate the mechanisms linking alcohol use with biological ageing and their interactions.

Several other limitations need to be addressed when interpreting these findings. First, this study relied on self-reported measures of alcohol use that might be inaccurate due to response biases. Whereas there are traditionally few alternatives, the validation of approaches such as estimation of alcohol drinking via the generation of a composite score for alcohol use from DNAm data^19,51^ or other biological data (e.g. liver enzyme levels as investigated in association with EAA previously^17^) could help to overcome the need to rely on self-reported measures. Second, although we investigated the associations between problematic alcohol use (AUDIT-P) and brain ageing, we were unable to conduct a similar evaluation in association with DNAm ageing measures. Additionally, different drinking patterns may be important. It was recently suggested that the relationship between educational attainment and adverse health outcomes is mediated by specific patterns of alcohol use, such as binge drinking, rather than total alcohol consumption.^52^ Thus, the focus on overall alcohol consumption in the present study might preclude the detection of a causal relationship. Finally, we investigated the associations between alcohol use and brain ageing or blood DNAm ageing in two separate large cohorts. It would be of interest in future studies to investigate the associations between alcohol use and the two types of biological ageing measures in the same individuals and evaluate the relationship between brain and DNAm ageing.

To conclude, in one of the largest studies on the relationship between alcohol use and biological ageing to date, and a first investigation of the causal relationship between the two using MR, we report a consistent association between higher levels of alcohol consumption and accelerated biological ageing. The present study found very limited evidence that the diagnosis of AUD might be causally linked to accelerated brain ageing; however, these results need to be interpreted with caution as they are driven by an outlier variant with unknown biological function and there is some limited evidence of potential reverse causation. Using two-sample MR, we additionally found no evidence for a causal link between alcohol consumption levels and biological ageing indicated by brain or DNAm ageing measures. The positive phenotypic associations between alcohol consumption and brain and epigenetic ageing add to the body of literature suggesting that alcohol use is associated with biomarkers predicting early ageing and mortality; however, the precise nature of this relationship remains to be identified in future studies.

## Supplementary Material

Supplementary Figure

Supplementary Table

## Data Availability

According to the terms of consent for GS:SFHS, access to data must be reviewed by the GS Access Committee (access@generationscotland.org). Details concerning access to UK Biobank data can be found in the link provided: https://www.ukbiobank.ac.uk/principles-of-access/. Summary statistics from GWAS of AgeAccelPheno and AgeAccelGrim are available here: https://doi.org/10.7488/ds/2834. Summary statistics from GWAS of brain age acceleration are available here: https://doi.org/10.7488/ds/2956.
