# Supplementary material for: Associations between alcohol use and accelerated biological ageing
Source: Addict Biol. Author manuscript; Available in PMC 2023 Feb 24. (PMC7614236; doi:10.1111/adb.13100)
Supplement: Supplementary Figure [file EMS169173-supplement-Supplementary_Figure.docx]

# Supplementary Figures
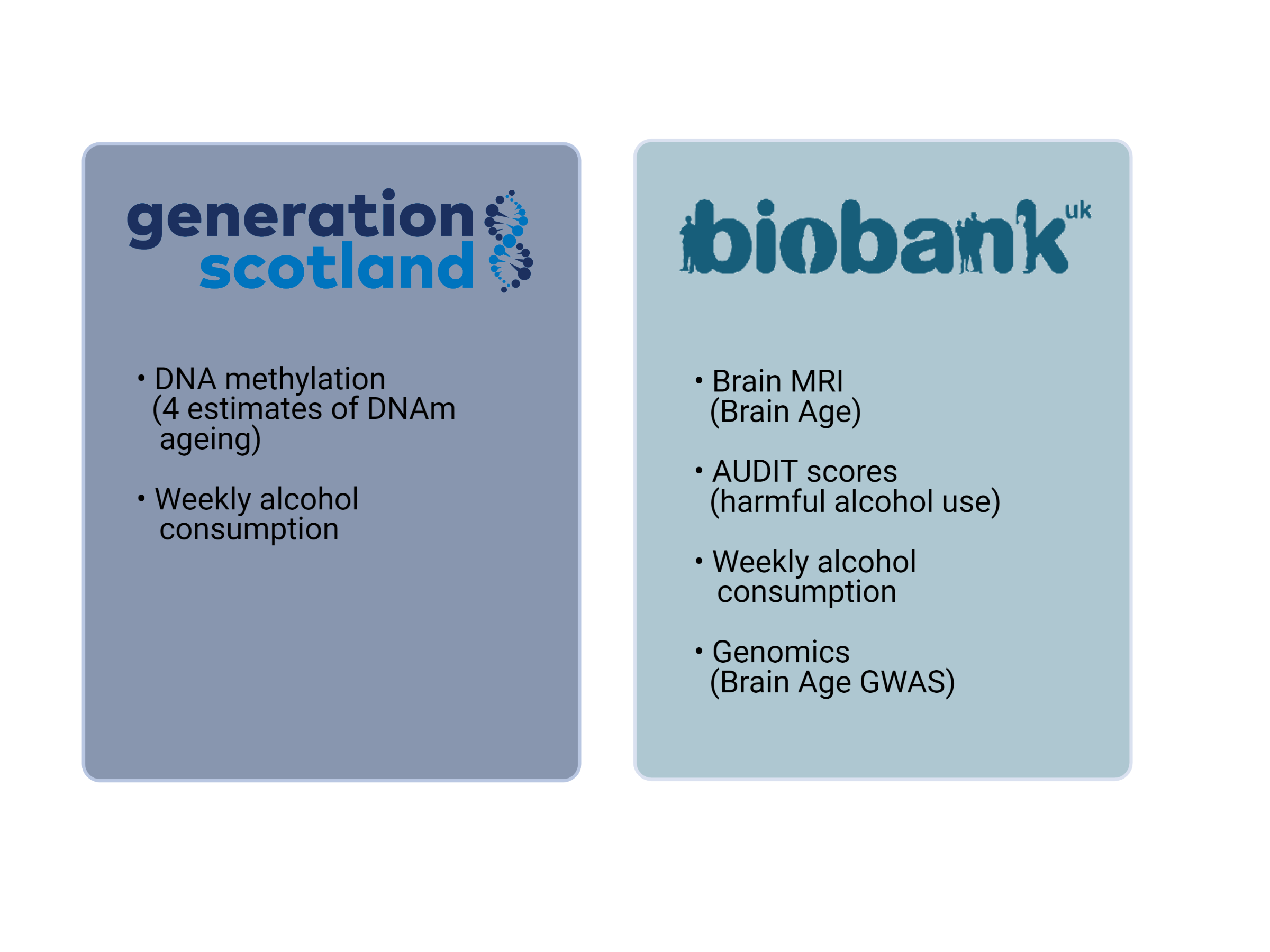


**Figure S1.** Graphical representation of the samples and data available in UK Biobank and The Generation Scotland: Scottish Family Health Study cohorts for the analyses conducted in the current study.


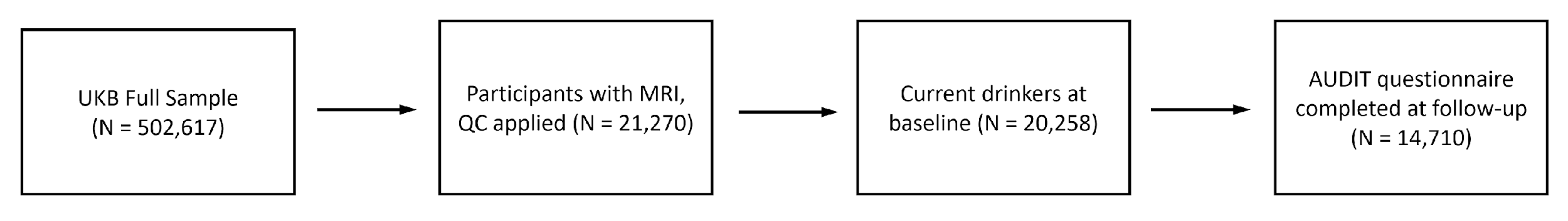


**Figure S2.** Flowchart showing sample selection in UKB for the present study. At the time of analysis, the number of participants in UKB totalled N=502,617 and of these, the latest neuroimaging release consisted of N=21,270 after quality control (QC; see main text for details of QC protocol). Of these, the present study included N=20,258 individuals who reported being current drinkers. A subset of UKB participants completed an online follow-up including the AUDIT questionnaire. Of the present sample of current drinkers with available MRI data, N=14,710 had completed the AUDIT questionnaire.

**
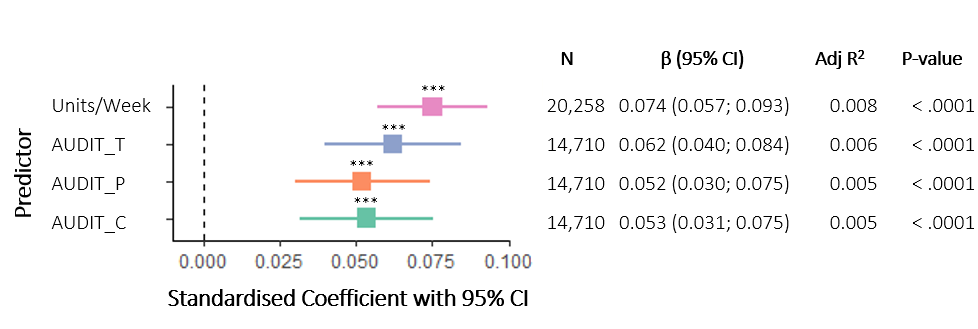
**

**Figure S3.**  Alcohol use is associated with advanced brain age. Linear regression models predicting residual brain age from AUDIT-C, AUDIT-P, AUDIT-T and alcohol units, in current drinkers adjusted for smoking status and age of completion of full-time education. Plot shows standardised 𝛃 coefficients with 95% confidence intervals. CI = confidence interval.


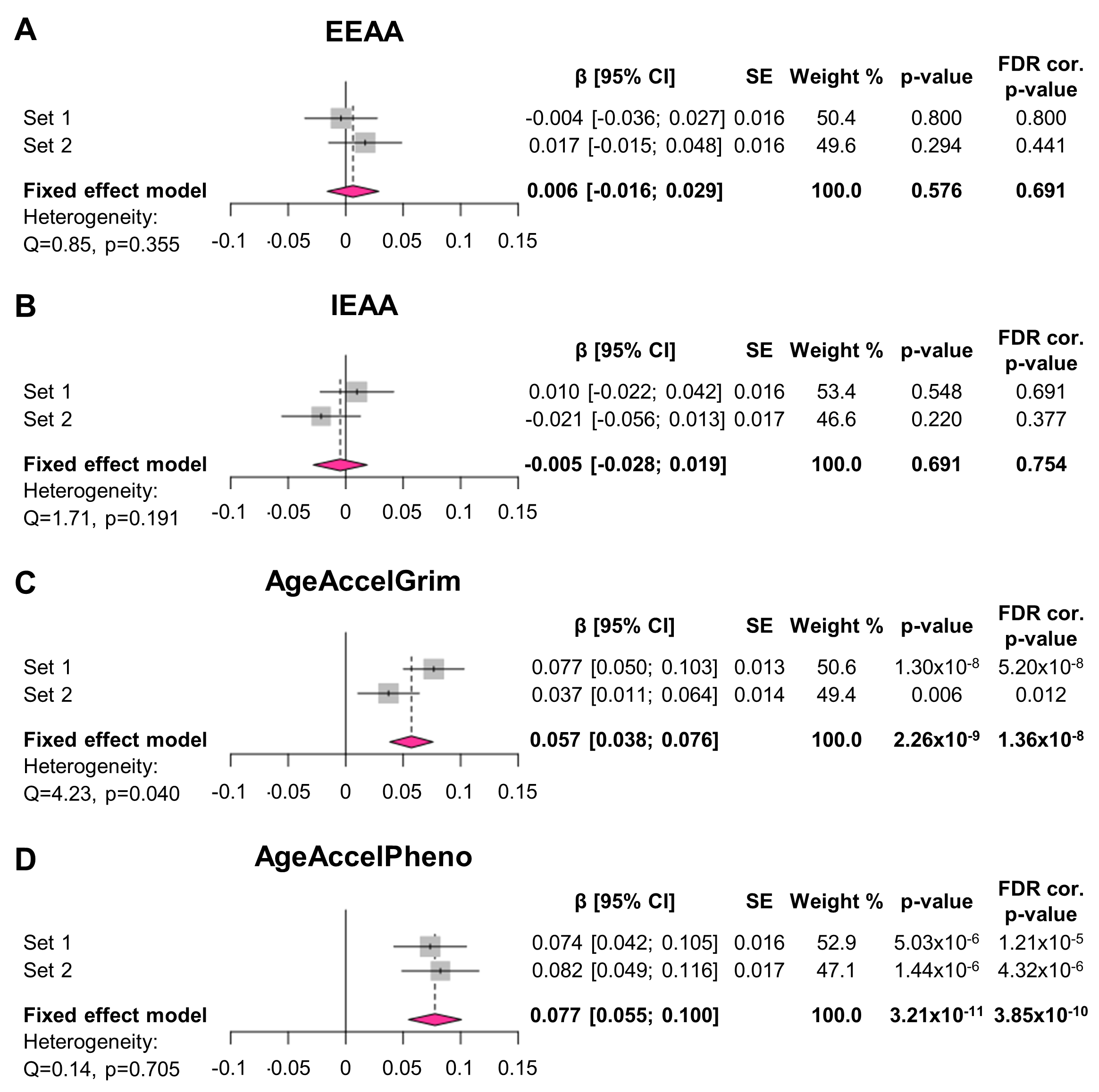


**Figure S4.** Alcohol consumption is associated with two measures of advanced epigenetic age. Effects of alcohol consumption (units/week) on (A) EEAA, (B) IEAA, (C) AgeAccelGrim and (D) AgeAccelPheno. Values on forest plot indicate standardised 𝛃 with 95% confidence intervals. Models are adjusted for sex, BMI, pack years, and years of education in sets 1 and 2, and relatedness in set 1 by fitting pedigree information as a random effect in general linear mixed models using advanced restricted maximum likelihood (ASReml) method. Fixed effect inverse variance weighted meta-analysis was applied using R package *meta* to combine the standardised coefficient estimates in set 1 and set 2. FDR correction was applied across all models in sets 1 and 2, and all meta-analysis models (12 models in total). Sample size: n=4132 in set 1, n=3691 in set 2 (n=7823 included in meta-analyses). EEAA = extrinsic epigenetic age acceleration, IEAA = intrinsic epigenetic age acceleration, SE = standard error, CI = confidence interval, FDR = false-discovery rate.


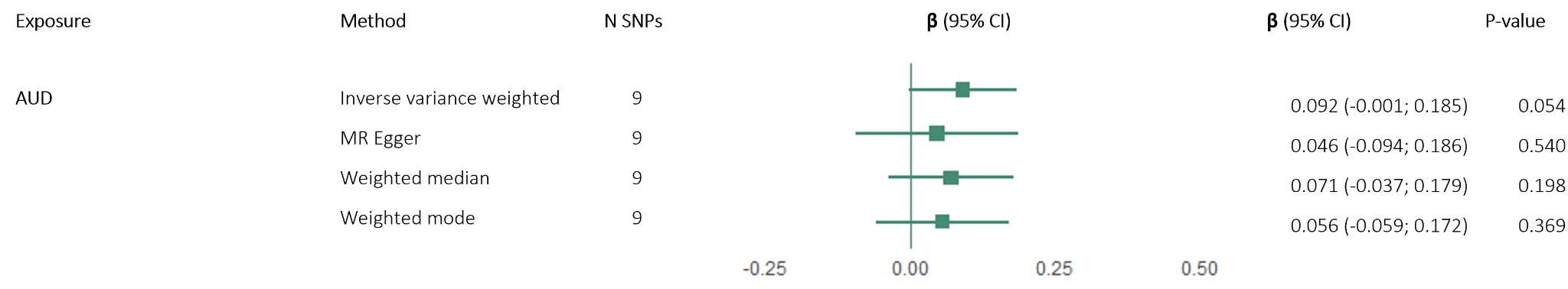


**Figure S5.** Two sample Mendelian randomisation of AUD on brain age. Results of Mendelian randomisation analysis with the outlying SNP rs570436 (Q=4.854, p=0.028, Radial MR) removed from the main analysis (Figure 4). Data on the genetic association with AUD was extracted from Kranzler et al^22^. Summary statistics for these SNPs were extracted from a novel GWAS of Brain Age (see Methods). N SNP=number of SNPs included in the MR analysis. No further sensitivity analysis was applied here.


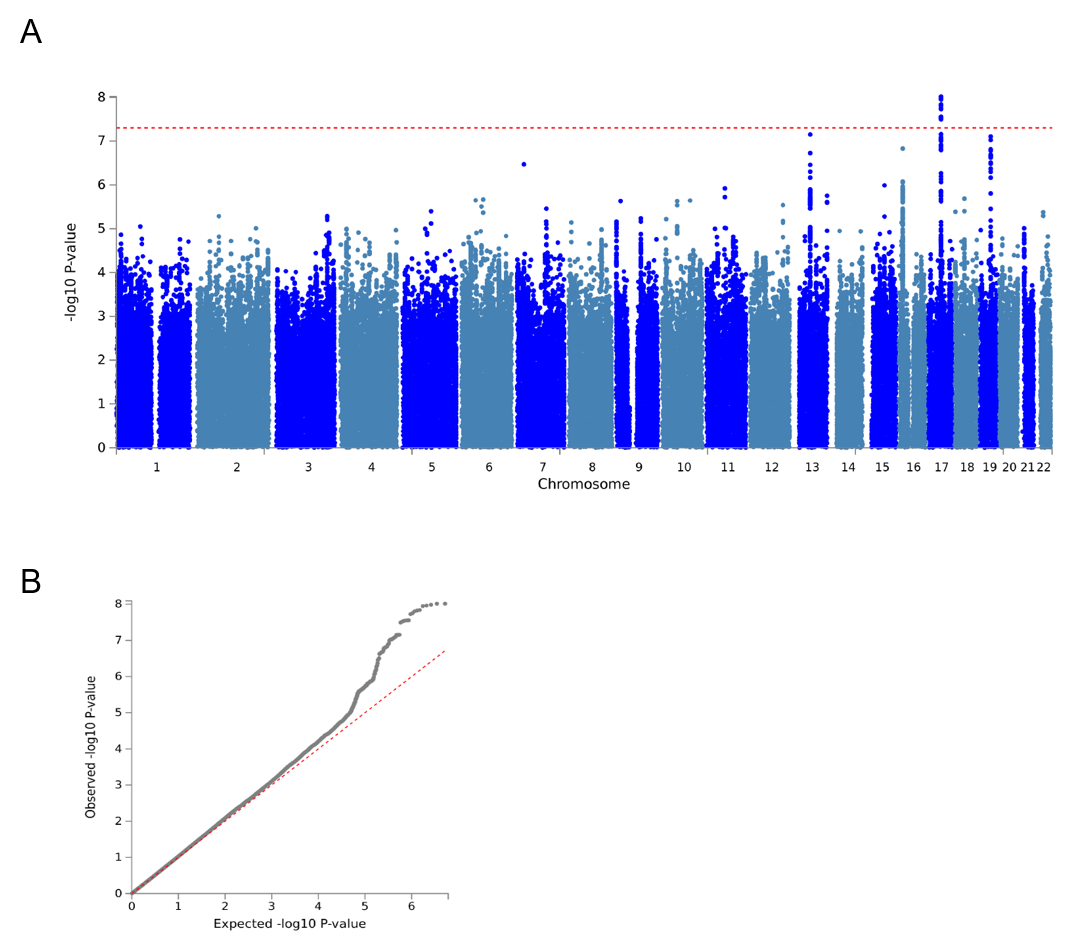


**Figure S6.** SNP-based Manhattan (A) and QQ (B) plots for GWAS of Brain Age, conducted in a subset of UKB (n = 16,133). A. Three genome-wide significant SNPs were identified by FUMA.
